# Rapid spheroid assays in a 3-dimensional cell culture chip

**DOI:** 10.1186/s13104-021-05727-0

**Published:** 2021-08-13

**Authors:** Jia Lin Teh, Siti Fairus Abdul Rahman, Gregory Domnic, Lengishwarra Satiyasilan, Nelson Jeng Yeou Chear, Darshan Singh, Nethia Mohana-Kumaran

**Affiliations:** 1 School of Biological Sciences, Universiti Sains Malaysia, 11800 Penang, Malaysia; 2 Centre for Drug Research, Universiti Sains Malaysia, 11800 Penang, Malaysia

**Keywords:** Spheroids, Nasopharyngeal carcinoma, 3D cell culture chip, Cisplatin, Mitragyna alkaloid, Paynantheine, Bovine collagen I

## Abstract

**Objective:**

The spheroid model provides a physiological platform to study cancer cell biology and drug sensitivity. Usage of bovine collagen I for spheroid assays is costly especially when experiments are conducted in 24-well plates, as high volume of bovine collagen I is needed. The aim of the study was to downsize spheroid assays to a microfluidic 3D cell culture chip and compare the growth, invasion and response to drug/compound of spheroids embedded in the 3D chip to spheroids embedded in 24-well plates.

**Results:**

Spheroids generated from nasopharyngeal carcinoma cell line HK-1 continuously grew and invaded into collagen matrix in a 24-well plate. Similar observations were noticed with spheroids embedded in the 3D chip. Large spheroids in both 24-well plate and the 3D chip disintegrated and invaded into the collagen matrix. Preliminary drug sensitivity assays showed that the growth and invasion of spheroids were inhibited when spheroids were treated with combination of cisplatin and paynantheine at high concentrations, in a 24-well plate. Comparable findings were obtained when spheroids were treated with the same drug combination in the 3D chip. Moving forward, spheroid assays could be performed in the 3D chip in a more high-throughput manner with minimal time and cost.

**Supplementary Information:**

The online version contains supplementary material available at 10.1186/s13104-021-05727-0.

## Introduction

Nasopharyngeal carcinoma (NPC) manifests beneath the nasopharyngeal mucosa or within the pharyngeal recess, known as the Fossa of Rosenmüller [1, 2]. NPC is responsible for more than 100,000 new cases and 72,987 deaths in 2018 [3]. Deeper understanding of the molecular mechanisms of NPC has led to testing of targeted therapies, immunotherapies and combination of these therapies with chemotherapeutic drugs.

Sensitivity of cancer cells to potential therapeutic agents are first evaluated in monolayer culture. Monolayer culture is economical, high-throughput and provides first-hand information on sensitivity of cells to drugs tested [4]. However, the model does not mimic the behaviour and microenvironment of tumour cells in vivo [4, 5]*.* The spheroid model recapitulates the properties of tumors in vivo such as cellular heterogeneity, microenvironment, cell–cell interactions, cell-extracellular matrix (ECM) interactions, growth kinetics, gene expression and drug resistance mechanisms [4, 6, 7]. Thus, the spheroid model provides a more physiological platform for drug screening [7–9].

Thus far, we have conducted spheroid assays in 24-well plates as described by Abdul Rahman et al.[10]. This method described in Abdul Rahman et al., was a modification of the method described in Smalley et al.[11]. Although the 24-well plate format allows different concentrations of drugs (e.g. double or triple drug combinations) to be tested, it is not amenable for testing multiple cancer cell lines and large collections of drugs at one time. The 24-well plate is coated with collagen matrix to re-create the microenvironment that tumours encounter in vivo. The main ingredient of the collagen matrix is bovine collagen I. The collagen type I is the major extra-cellular protein found in human organs. Given that the collagen type I is available in abundance in the bovine collagen I, it is preferred in our experiments [12]. However, spheroid assays conducted in 24-well plates require high volume of collagen matrix. Hence, high volume of bovine collagen I is needed which is costly. Moreover, the set-up of experiments is laborious and time-consuming.

We employed the 3D cell culture chip (hereafter will be referred to as ‘3D chip’) to conduct spheroid drug sensitivity assays. The 3D chip is amenable to test multiple cancer cell lines, subjected to different drug combinations and concentrations. The spheroids are embedded in collagen matrix, recapitulating the tumour microenvironment in vivo. The 3D chip requires 10 times less bovine collagen I to prepare the collagen matrix and the experiment set-up takes less than an hour. Collectively, the 3D chip allows drug screening to be conducted with minimal cost and in a high-throughput manner without having to compromise tumour microenvironment.

In this pilot study, prior to conducting drug sensitivity assays, growth and invasion of HK-1 spheroids (spheroids generated from NPC cell line HK-1) embedded in the 3D chip and the 24-well plate were first compared. In order to ensure that the findings of drug sensitivity assays conducted in the 24-well plate is comparable to the findings in the 3D chip, HK-1 spheroids were treated with combination of cisplatin and paynantheine (a mitragyna alkaloid compound which was isolated and purified from kratom leaves—a local medicinal plant found in Malaysia) [13] in the 3D chip and the 24-well plate, simultaneously. Snapshots of HK-1 spheroids were taken every 24 h to monitor the effect of the drug combination on spheroid growth and invasion.

## Main text

### Methods

More detail steps on how to embed spheroids in the 3D chip are shown in Additional file 1.

#### Embedding HK-1 spheroids into collagen matrix

**Fig. 1:**
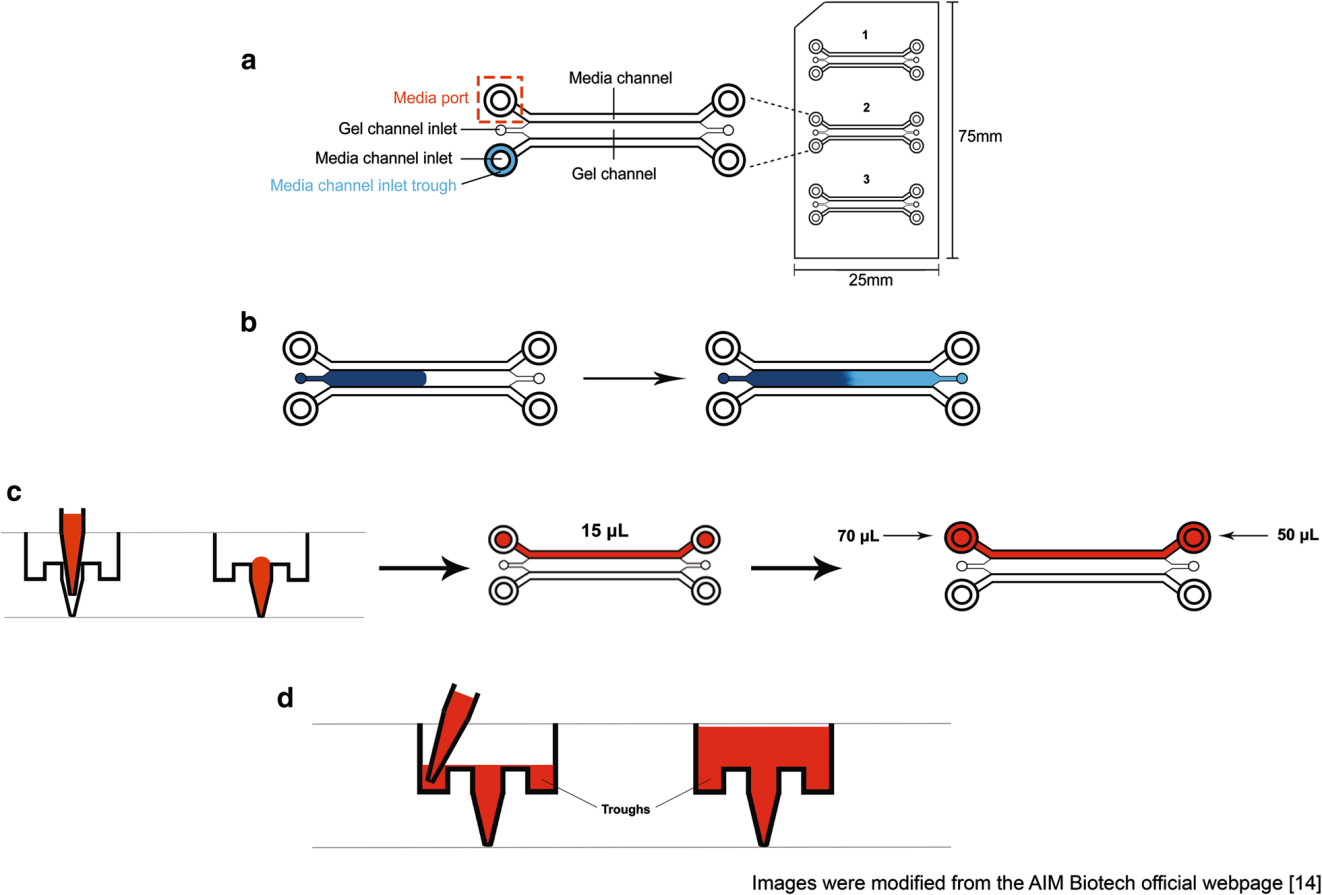
The 3D cell culture chip. **a** The design of the 3D cell culture chip (AIM Biotech). The chip is designed as a standard size microscope specimen slide and it consists of three columns. Each column comprises of a gel channel surrounded by two media channels. Spheroids were passed through the gel channels after resuspending the spheroids with collagen. Media channels were filled with either complete media or complete media plus drugs depending on the objectives of the experiment. **b** Suspension of collagen matrix with spheroids into the gel channels of the 3D chip. **c** Hydrating the media channel ports with media only or media + drug. **d** Replenishment of media or media + drug in the chip

The HK-1 cells were authenticated using the AmpFISTR profiling and obtained from the Molecular Pathology Unit, Institute for Medical Research (IMR), Malaysia. Spheroids were generated as described in Abdul Rahman et al. [10]. Spheroids generated from 800 to 1000 HK-1 cells grow into appropriate sizes to be embedded into the 3D chip (AIM Biotech, Singapore) (Fig. 1a). Media were completely removed from the microcentrifuge tubes and the spheroids were re-suspended with 11 μL of collagen mix. Spheroids were embedded into the 3D chip as described [14]. Five microliters of collagen mix containing spheroids were injected into the 3D chip’s gel inlet from one end and stopped when the collagen reached the middle of the channel (Fig. 1b—refer to the dark blue region). The remaining 5 μL of the collagen mix containing spheroids were injected into the other end of the gel inlet until the collagen front merges (Fig. 1b—refer to the light blue region). The collagen mix containing spheroids must be carefully and slowly injected into the gel inlets to avoid formation of bubbles. The 3D chip was transferred into a humidified chamber and the chamber was covered with a thin sheet of aluminium foil. The chip was incubated in a humidified incubator at 37 °C with 5% CO_2_ for 30 min to allow solidification of the collagen matrix. The 3D chip’s media channel was hydrated with 15 μL of media + drug (Fig. 1c). One end of the media channel port was filled with 70 μL of media + drug and the opposite end with 50 μL of complete media + drug to create a pressure gradient for smooth and continuous flow of the liquid. The same steps were repeated for the adjacent media channels (Fig. 1c). The chip was placed in a humidified chamber and the chamber was covered with a thin sheet of aluminium foil. The chip was incubated in a humidified incubator at 37 °C with 5% CO_2_. As for spheroid assays conducted in 24-well plate, spheroids were embedded as described [10–12]. Snapshots of spheroids growth and invasion in both 24-well plate and 3D chip were taken every 24 h using a phase contrast microscope and images were analysed using Image J [15].

#### Replenishment of media and drug

Media and drug must be replenished every 72 h for long-term experiments to avoid formation of air columns in the gel channels and complete dryness of the channels in the 3D chip. Media in the media ports were aspirated from the troughs (Fig. 1d). Media and drug were replenished in the media channel ports using the same volumes as described in the previous section. The 3D chip was placed in a humidified chamber and the chamber was covered with a thin sheet of aluminium foil. The 3D chip was incubated in a humidified incubator at 37 °C with 5% CO_2_. Similarly, media and drug were replenished in the 24-well plate every 72 h for long-term experiments.

#### Quantification of spheroid growth

Spheroid growth were analysed using the Image J software [15]. More detailed steps on spheroid growth quantification can be found in Additional file 2.

### Results

#### Spheroid assays conducted in the 3D chip were comparable to spheroid assays conducted in a 24-well plate

**Fig. 2:**
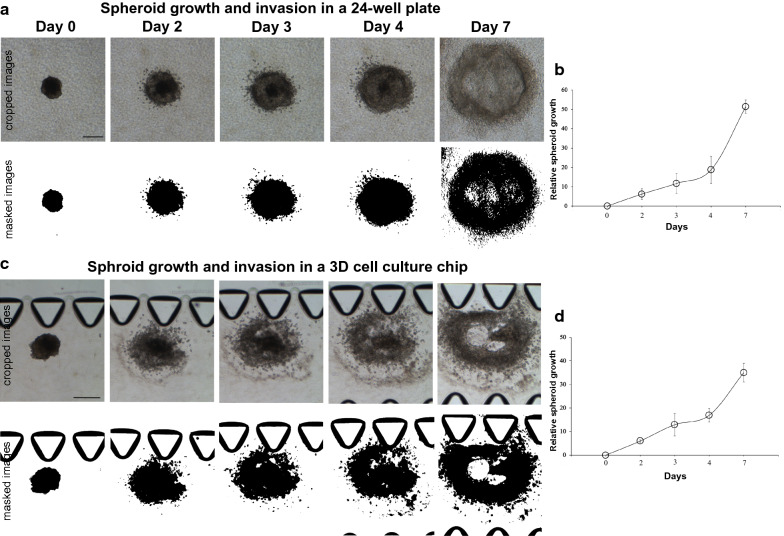
Growth and invasion of HK-1 spheroids in the 24-well plate and in the 3D chip. **a** Growth and invasion of an untreated HK-1 spheroid embedded into collagen matrix in the 24-well plate. Spheroid continuously grew and invaded into the collagen matrix. Images were masked using Image J to show clarity of the growth and invasion of the spheroids. Image crop factor: 500 × 500. Images were resized to 120 × 120 pt on Adobe Illustrator. (Size bar: 200 μm). **b** Graphs show corresponding quantification of spheroid growth in the 24-well plate for the days shown, n = 2 spheroids. Error bars indicate standard errors. **c** Growth and invasion of an untreated HK-1 spheroid embedded into collagen matrix in the 3D chip. Spheroid continuously grew and invaded into the collagen matrix. Images were masked using Image J to show clarity of the growth and invasion of the spheroids. Image crop factor: 798 × 900. Images were resized to 120 × 140 pt on Adobe Illustrator. (Size bar: 200 μm). **d** Graphs show corresponding quantification of spheroid growth in the 3D chip for the days shown, n = 2 spheroids. To eliminate the different spheroid sizes on day 0, spheroid growth on day 0 was set at zero (spheroid growth on day 0—spheroid growth on day 0). The relative spheroid growth for each day was determined by subtracting the spheroid growth obtained on a particular day with the spheroid growth obtained at day 0 (for example, spheroid growth on day 2—spheroid growth on day 0). Error bars indicate standard errors

Given that this is the first time we were using the 3D chip, to perform experiments with the HK-1 spheroids, the growth and invasion of the spheroids were tested in the 24-well plate and in the 3D chip simultaneously. This assessment was to ensure that there were no discrepancies in the ability of the HK-1 spheroids to grow and invade in the 3D chip as compared to in the 24-well plate. Snapshots of the HK-1 spheroids, in the 24-well plate, showed that the spheroids continually grew and invaded into the collagen matrix (Fig. 2a). Quantification of relative spheroid growth area demonstrate that spheroids were viable throughout the duration of the experiment, as the spheroids grew in size to cover more area of the collagen matrix, over 7 days (Fig. 2b). Similar observations were noticed with spheroids embedded in the 3D chip (Fig. 2c, d). Spheroids in both the 24-well plate and the 3D chip disintegrated and invaded into the collagen matrix over time (Fig. 2a and c—see the holes in the spheroids that were not masked by the imaging software). It appears that this disintegration of cells occurred more rapidly in spheroids embedded in the 3D chip (by day 3) compared to in the 24-well plate (Fig. 2a and c).

**Fig. 3:**
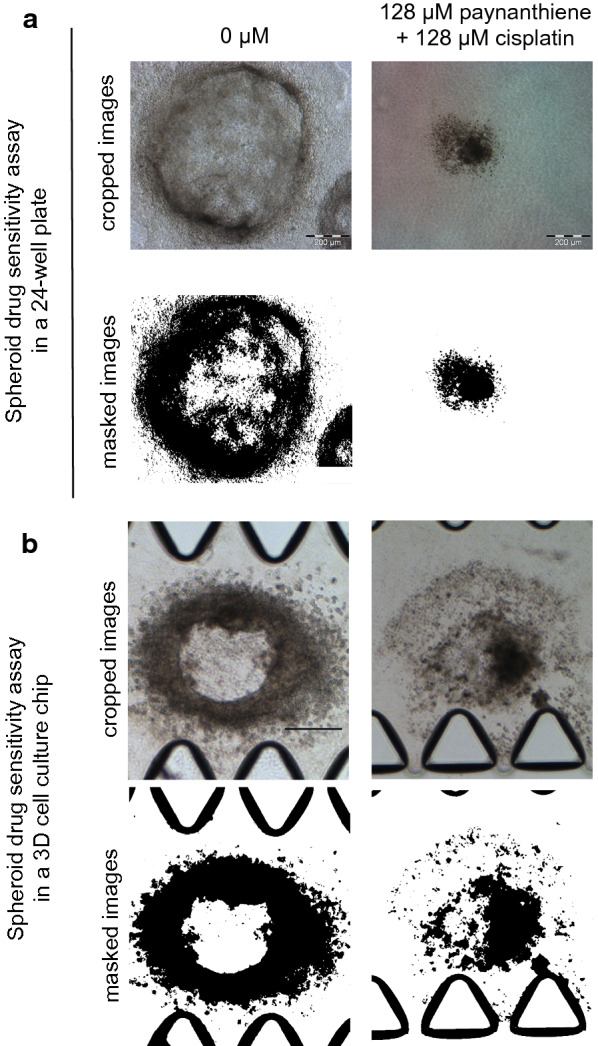
The effect of combination of cisplatin and paynantheine on the growth and invasion of HK-1 spheroids. Spheroids embedded in the 24-well plate and in the 3D chip were treated with combination of cisplatin and paynantheine at high concentrations. **a** The drug combination inhibited the growth and invasion of the spheroid in the 24-well plate. Images were masked using Image J to show clarity of the growth and invasion of the spheroids. Image crop factor: 900 × 900. Images were resized to 120 × 102 pt on Adobe Illustrator. (Size bar: 200 μm). **b** The drug combination inhibited the growth and invasion of the spheroid in the 3D chip. Images were masked using Image J to show clarity of the growth and invasion of the spheroids. Image crop factor: 798 × 900. Images were resized to 120 × 140 pt on Adobe Illustrator. (Size bar: 200 μm)

In order to ensure that the findings of the spheroid drug sensitivity assay conducted in the 3D chip is comparable to the findings of the same assay conducted in a 24-well plate, the spheroid drug sensitivity was conducted in the 3D chip and in the 24-well plate, simultaneously. The spheroid drug sensitivity assay performed in the 24-well plate often employs spheroids generated from 5000 cells. Although the HK-1 spheroids generated from 800 to 1000 cells produced more appropriate spheroid sizes for the 3D chip, the spheroids generated from 5000 HK-1 cells were embedded in the 3D chip in order to avoid discrepancies in data. The spheroids embedded in the chip and in the 24-well plate were treated with combination of cisplatin and paynantheine at high concentrations. The drug combination inhibited spheroid growth and invasion in the 24-well plate (Fig. 3a and Additional file 3a). Similar drug combination effect was observed with spheroid embedded in the 3D chip (Fig. 3b and Additional file 3b) demonstrating that the findings in the 3D chip and 24-well plate were analogous.

## Discussion

The spheroid model provides a physiological platform for drug screening as it closely mimics tumours in vivo [4]. Spheroid assays conducted in 24-well plates require high volume of bovine collagen I which is costly and therefore drug sensitivity assays cannot be performed in a high-throughput manner. The 3D chip only requires a small amount of bovine collagen I, thus reducing the cost of conducting spheroid assays considerably. Given that there were lack of optimized protocol for embedding and conducting drug sensitivity assays with spheroids generated from NPC cell lines, in the 3D chip, the discussion provided are primarily based on our experience with the 3D chip.

Injecting collagen matrix together with spheroids into the gel channel inlets is a crucial step. The collagen matrix with spheroids is first injected from one gel inlet until the gel front reaches the middle of the channel. The remaining collagen matrix is injected slowly and carefully from the opposite gel inlet and must be stopped immediately once the collagen matrix meets the gel front. Failure to do so, may create a strong pressure and may push the injected spheroids back to the gel channel inlet. Moreover, slanting the 3D chip while transferring it to the incubator must be avoided. At this point the collagen matrix is still in its aqueous form and the slant may cause spheroids to flow back and get trapped in the gel inlets (Additional file 3c).

The chip is able to accommodate large spheroids generated from 5000 HK-1 cells. However, smaller spheroids generated from 800 to 1000 cells are preferred due to several reasons. Large spheroids generated from 2000 or 5000 cells have the tendency to embed closer or trapped between the ‘teeth’ like structures of the gel channel borders (Additional file 3d, e). These ‘teeth’ like structures cause obstacles during quantification of spheroid growth and invasion. We speculate that larger spheroids probably resist flow during injection of the collagen matrix into the gel channel, which in turn increases the tendency of these spheroids to embed closer to the ‘teeth’ like structures of the gel channels.

Large spheroids should be avoided in experiments exceeding more than three days. Although at times large spheroids are embedded away from the ‘teeth’ like structures, during long-term experiments, these spheroids eventually grow and invade and fully occupy the width of the gel channel and touch the ‘teeth’ like structures (Additional file 3f). Again, this may affect the quantification of spheroid growth and invasion.

The 3D chip when it is not covered after placing it in a humidity chamber, results in formation of air columns in the gel channels and complete dryness of the channels. Failure to maintain humidity inside the 3D chip chambers results in spheroids not receiving sufficient nutrients. For example, in an experiment where humidity was not maintained properly, growth and invasion of spheroids were hampered (Additional file 3 g, h—see spheroids generated from 800 and 2000 HK-1 cells). The humidified chamber when loosely covered with a thin sheet of aluminium foil, improved humidity inside the 3D chip chamber. Maintenance of humidity inside the 3D chip chamber slowed down the evaporation rate of media and ensured continuous capillary flow of media into the gel channels which housed the spheroids. Spheroids continuously grew and invaded into the collagen matrix (Additional file 3i—see spheroid generated from 5000 HK-1 cells). As a note of caution, large spheroids may not be ideal for experiments exceeding three days for the reasons discussed above. Spheroids generated between 800 and 1000 cells may be more ideal for long-term experiments provided humidity in the 3D chip chambers are maintained.

## Limitations

One limitation of the 3D chip is that there are only three columns per chip. Multiple 3D chips have to be used to test different drug combination concentrations. The effect of combination of cisplatin and paynantheine at lower doses were not interrogated in this study. Ongoing studies in the laboratory are investigating various drug combination doses of cisplatin and paynantheine in order to study the effect of these various combinations on NPC cell proliferation.

## Supplementary Information

**Additional file 1**. Steps to embed spheroids in the 3D chip.

**Additional file 2**. Steps to quantitate spheroid growth.

**Additional file 3**. Quantification of relative spheroid size in the presence and absence of combination treatment of paynanthiene and cisplatin.

## Data Availability

Not applicable.

## Abbreviations

NPC: Nasopharyngeal carcinoma
ECM: Extracellular matrix
3D: 3-Dimensional
