# Supplementary material for: Rapid spheroid assays in a 3-dimensional cell culture chip
Source: BMC Res Notes. 2021 Aug 13;14:310. doi: 10.1186/s13104-021-05727-0 (PMC8361632; doi:10.1186/s13104-021-05727-0)
Supplement: Supplementary file 2 — Additional file 2. Steps to quantitate spheroid growth. [file 13104_2021_5727_MOESM2_ESM.docx]

**Additional file 2**

**Quantification of spheroid growth**

Spheroid growth were analysed using the Image J software [15]. The following were the steps followed for quantifying spheroid using the Image J software. Step 1: The spheroid image in tiff format was opened in the Image J software (Go to Image > Type > 8-bit). Step 2: The brightness and contrast of the images was auto adjusted (Go to Image > Adjust > Brightness/Contrast > Auto > Apply). Step 3: Set threshold (Go to Image > Adjust > Threshold > Adjust the threshold to fill the spheroid area manually using the sliders > Apply). Step 4: Create mask (Go to Edit > Selection > Create Mask). Step 5: Analyze spheroid area [Go to Analyze > Analyze particle (set Size: 0-infinity, Show: Outlines, Choose: Display results, Clear results, Summarize and Click OK]. Step 6: The area fraction of the spheroid can be obtained from the “Results” window and the total area and percentage of area can be obtained from the “Summary” window. The relative spheroid area was calculated using the formula: Area fraction/Total area X Area percentage
