# Supplementary material for: Rapid spheroid assays in a 3-dimensional cell culture chip
Source: BMC Res Notes. 2021 Aug 13;14:310. doi: 10.1186/s13104-021-05727-0 (PMC8361632; doi:10.1186/s13104-021-05727-0)
Supplement: Supplementary file 3 — Additional file 3. Quantification of relative spheroid size in the presence and absence of combination treatment of paynanthiene and cisplatin. [file 13104_2021_5727_MOESM3_ESM.pdf]

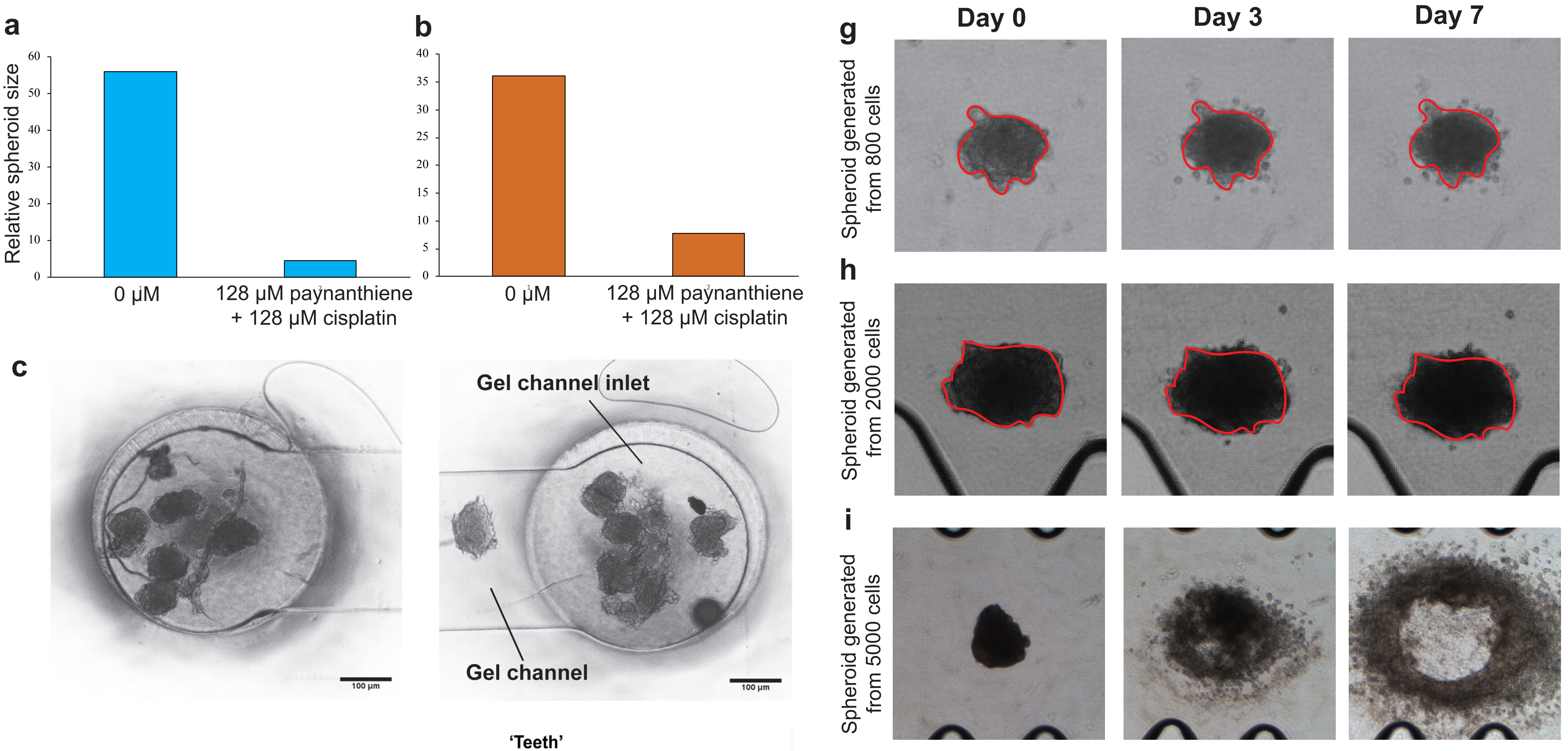

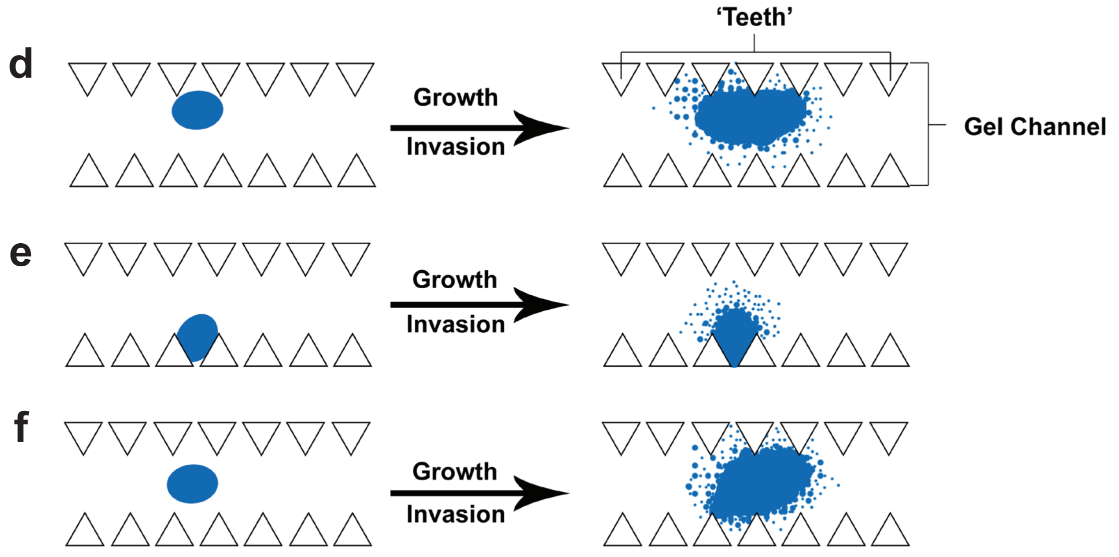

Additional file 3: Quantification of relative spheroid size in the presence and absence of combination treatment of paynanthiene and cisplatin. Graphs show corresponding quantification of treated and untreated spheroids shown in figure 3, embedded in (a) 24-well plate and (b) 3D chip, using image analysis. (c) The HK-1 spheroids trapped in the gel channel inlets of the 3D chip and failed to enter the gel channel. Illustrations of issues encountered when large spheroids are embedded into the gel channels of the 3D chip. (d) Spheroid (shown in blue) is embedded close to the 'teeth' like structures of the gel channel border. (e) Spheroid is trapped in between 'teeth' like structures of the gel channel border. (f) Spheroid embedded at the centre of the channel grows and invade towards the 'teeth' like structures. Snapshots of growth and invasion of spheroids generated from (g) 800; (h) 2000 and (i) 5000 HK-1 cells in the 3D chip. Media channels of the chip were dry due to media evaporation by day 3. Failure to maintain humidity in the chip columns hampered the growth and invasion of the HK-1 spheroids generated from 800 cells. A red outline was drawn on the "Day 0" spheroid image and the outline was copied and pasted on every snapshot. There were very few cells which invaded into the collagen matrix. HK-1 spheroids generated from 2000 cells also exhibited negligible growth. Maintenance of humidity in the columns of the chip embedded with spheroids generated from 5000 HK-1 cells, supported continuous spheroid growth and invasion into the collagen matrix as there were sufficient availability of media. Crop factor of images shown in the first two rows : 200 x 200. Images were resized to 150 x 150 pt on Adobe Illustrator. Crop factor of images shown in the last row: 700 x 700. Images were resized to 150 x 150 pt on Adobe Illustrator. The crop factor for these images were larger to show the growth and invasion of the spheroids with better clarity. (Size bar: 200 μm)
